# Structural and functional analysis of *Oceanobacillus iheyensis* macrodomain reveals a network of waters involved in substrate binding and catalysis

**DOI:** 10.1098/rsob.160327

**Published:** 2017-04-26

**Authors:** Rubén Zapata-Pérez, Fernando Gil-Ortiz, Ana Belén Martínez-Moñino, Antonio Ginés García-Saura, Jordi Juanhuix, Álvaro Sánchez-Ferrer

**Affiliations:** 1 Department of Biochemistry and Molecular Biology-A, Faculty of Biology, Regional Campus of International Excellence ‘Campus Mare Nostrum’, University of Murcia, Campus Espinardo, 30100 Murcia, Spain; 2 CELLS-ALBA Synchrotron Light Source, 08290 Barcelona, Spain; 3 Murcia Biomedical Research Institute (IMIB-Arrixaca), 30120 Murcia, Spain

**Keywords:** macrodomains, crystal structure, mutational analysis, kinetic characterization, catalytic mechanism

## Abstract

Macrodomains are ubiquitous conserved domains that bind or transform ADP-ribose (ADPr) metabolites. In humans, they are involved in transcription, X-chromosome inactivation, neurodegeneration and modulating PARP1 signalling, making them potential targets for therapeutic agents. Unfortunately, some aspects related to the substrate binding and catalysis of MacroD-like macrodomains still remain unclear, since mutation of the proposed catalytic aspartate does not completely abolish enzyme activity. Here, we present a functional and structural characterization of a macrodomain from the extremely halotolerant and alkaliphilic bacterium *Oceanobacillus iheyensis* (OiMacroD), related to hMacroD1/hMacroD2, shedding light on substrate binding and catalysis. The crystal structures of D40A, N30A and G37V mutants, and those with MES, ADPr and ADP bound, allowed us to identify five fixed water molecules that play a significant role in substrate binding. Closure of the β6–α4 loop is revealed as essential not only for pyrophosphate recognition, but also for distal ribose orientation. In addition, a novel structural role for residue D40 is identified. Furthermore, it is revealed that OiMacroD not only catalyses the hydrolysis of *O*-acetyl-ADP-ribose but also reverses protein mono-ADP-ribosylation. Finally, mutant G37V supports the participation of a substrate-coordinated water molecule in catalysis that helps to select the proper substrate conformation.

## 1. Introduction

Post-translational protein modifications play a key role in many cell processes. Among them, the transfer of single or multiple ADP-ribose (ADPr) moieties from NAD^+^ to specific residues on a target protein is important in the regulation of DNA repair, transcription, telomere length, cell differentiation and proliferation, inflammatory and immune responses, unfolded protein response, and cell death [1]. In this dynamic and transient process, several enzymes are involved. Protein modification is initiated by ADP-ribosyl transferases, a group that includes human poly-ADPr polymerases (hPARPs). Among these, only hPARP1, hPARP2 and tankyrases are able to endow target proteins with long branched chains of poly-ADPr (PAR), whereas the remaining hPARPs transfer a mono-ADPr (MAR) group [2]. Since irreversible poly-ADP-ribosylation is highly deleterious, leading to progressive neurodegeneration and early embryonic lethality [3], PAR modification can be reversed through poly-ADPr glycohydrolase (PARG) and ADP-ribosyl hydrolase 3 (ARH-3), giving rise to MARylated proteins [4]. The final protein-linked MAR residue is then processed by ARH1 (when linked to Arg) or by macrodomains (hMacroD1, hMacroD2, viral macrodomains and C6orf130/TARG1/OARD1) in the case of acidic residues. However, only OARD1 macrodomain is able to remove *en bloc* the whole PAR polymer [5].

**Figure 1.**
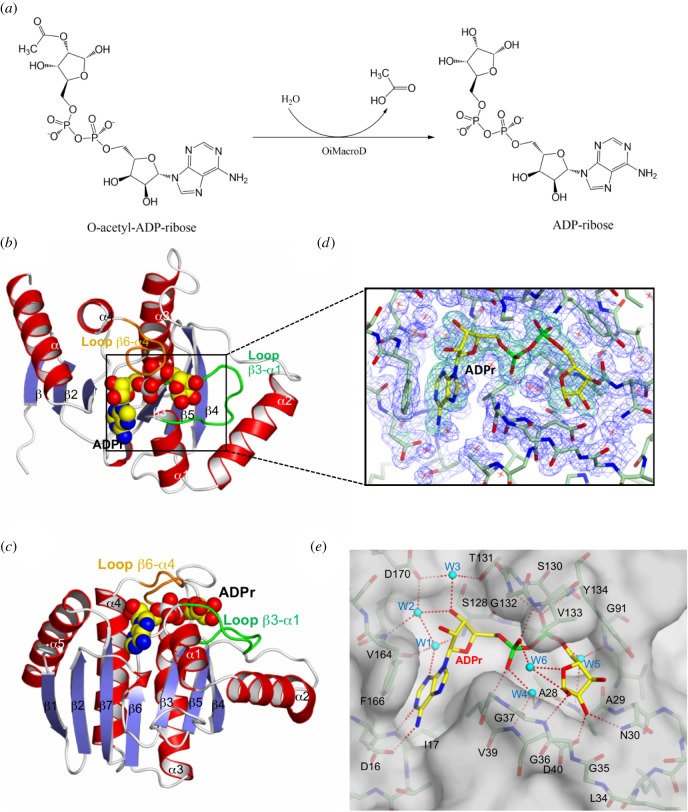
OiMacroD structure and ADPr binding site. (*a*) Reaction catalysed by MacroD-like macrodomains. (*b*,*c*) Orthogonal views of the OiMacroD subunit bound to ADPr (in space-filling). Key loops involved in substrate binding are coloured in green and orange, respectively. (*d*) *2Fo−Fc* ED map (1.0 σ; blue) is shown for all the elements, while the *Fo−Fc* omit ED map (2.5 σ; green) is only shown around the ADPr molecule. (*e*) Substrate cleft showing the interactions between ADPr, surrounding residues and structural water molecules (cyan). H-bonds are shown as red dashed lines. W3 was modelled as found in the G37V structure, since this water molecule in the OiMacroD-ADPr complex was replaced by a glycerol molecule coming from the cryoprotectant, with one oxygen atom mimicking the same interactions.

Macrodomains are ubiquitous and evolutionarily conserved modules (approx. 130–190 residues) that recognize ADPr derivatives in their free form or linked to proteins, including ADPr, PAR, ADP-ribose-1″phosphate and *O*-acetyl-ADPr (OAADPr) [6,7]. In addition to its role in ADP-ribosylation, some macrodomain family members, named MacroD-like proteins, including hMacroD1, hMacroD2 and *Escherichia coli* Ymdb, are also involved in the hydrolysis of products of NAD^+^-consuming enzymes, such as sirtuins [8–12]. These MacroD-like proteins catalyse the hydrolysis of OAADPr into ADPr and free acetate (figure 1*a*) [8]. Unlike these catalytic macrodomains, non-catalytic macrodomains (e.g. MacroH2A-like family) are only able to bind ADPr metabolites and ADP-ribosylated proteins [7]. Furthermore, in some cases, they are grouped in tandem in the same protein (e.g. hPARP9, hPARP14 and hPARP15) [13]. However, it is unclear how this globular domain has evolved from a binding to a catalytic domain, while causing minimal structural changes to the overall fold [1]. In addition, although the catalytic mechanism of macrodomains belonging to the OARD1-like family is known [5,14], the mechanism of the MacroD-like family has not been unequivocally defined. Two different catalytic mechanisms have been proposed for hMacroD1/D2 [8,9,15]. The most accepted model proposes a conserved aspartate as a base to deprotonate a nearby coordinated water molecule, facilitating hydrolysis of the ester bond [8,15]. However, the mutation of this aspartate residue does not manage to completely abolish the enzyme activity, and not all MacroD-like macrodomains possess such aspartate residue. The alternative model suggests a substrate-driven mechanism, in which a water molecule coordinated between the ADPr α-phosphate (Pα) and its distal ribose is activated by the Pα group for nucleophilic attack on the carbonyl carbon [9]. All the above-described open questions make further structural and biochemical characterization of macrodomains of great biological and biomedical interest [1]. More importantly, the intimate connections between macrodomains and the sirtuin and PARP family enzymes could make them a promising therapeutic target in some diseases (chronic lymphocytic leukaemia [14] and cancer [5]) or as antiparasitic drugs [12].

To expand our knowledge concerning the field of macrodomains, we have characterized *Oceanobacillus iheyensis* macrodomain (OiMacroD), the first to be described from the order *Bacillales*. The organism selected is an extremely halotolerant and alkaliphilic bacterium collected from a depth of 1050 m off the coast of Japan [16], which has attracted attention in the search for robust biocatalysts, as in other halotolerants [17], for solving the crystal structure of the prime example of a β-lactamase evolved in the absence of clinical selection [18], and for deciphering the catalytic mechanism of its galactarate dehydratase [19]. In addition, several studies on NAD^+^-related enzymes in *O. iheyensis* have been carried out [20,21]. In order to shed light on the correct catalytic mechanism, six OiMacroD crystal structures were solved, allowing us to determine a network of structural water molecules, which are largely responsible for coordinating the substrate. The active site point mutants, D40A and N30A, revealed the induced conformational movement carried out by the β6–α4 loop to correctly position the distal ribose for catalysis, stacking it with residue Y134. Finally, we show that OiMacroD and its mutants can reverse protein mono-ADP-ribosylation, G37V showing the lowest activity. The activity of this last mutant towards OAADPr also supports the involvement of a substrate-coordinated water molecule in catalysis, even though it is not close to the 2″-OH, suggesting a flexible catalytic mechanism involving different OAADPr isomers.

## 2. Results and discussion

### 2.1. OiMacroD is a member of the MacroD-like macrodomains class

OiMacroD gene was successfully cloned into pET28a and expressed in *E. coli* Rosetta 2 cells. A yield of 40 mg of pure protein per litre of culture after a simple two-step purification process was obtained (electronic supplementary material, figure S1). Its phylogenetic analysis revealed that, among the six different classes previously described for macrodomains [7], OiMacroD is included in the MacroD-type clade, together with other previously characterized MacroD-like macrodomains, such as hMacroD1 and hMacroD2 (electronic supplementary material, figure S2). The sequence and catalytic mechanism of these macrodomains differ from those of the ALC1-like family, which includes the human OARD1 macrodomain. The rest of the macrodomains in the tree are distributed in four additional clades. The H2A-like macrodomain clade clusters non-catalytic macrodomains, where PARP9, PARP14 and PARP15 are included, whereas the PARG-like clade groups enzymes involved in the degradation of PAR (electronic supplementary material, figure S2). Finally, the Macro2-type clade includes poorly studied macrodomain proteins, whereas the remaining clade shows viral proteins containing the active X-domain and also the SUD-M-like proteins, which contain both an X-domain and an M-domain [22] (electronic supplementary material, figure S2). This latter M-domain fails to bind to ADPr and interacts with G-quadruplexes [22].

### 2.2. A network of water molecules organizes the macrodomain active site

Three crystal structures of monomeric OiMacroD wild-type (WT) complexed with MES, ADPr or ADP were obtained at 1.9, 1.77 and 1.75 Å resolution, respectively (table 1). In addition, three structures corresponding to active site mutants (N30A, G37V and D40A) were obtained at 1.76, 1.35 and 2.0 Å resolution, respectively (table 1). The full-length OiMacroD structure (185 residues) has a single globular structure with approximate dimensions of 25 × 34 × 47 Å. The subunit has a mixed α/β fold, similar to that of other nucleotide-binding proteins, composed of a central seven-stranded mixed β-sheet (β1–β2–β7–β6–β3–β5–β4) and five α-helices (figure 1*b,c*). The five central strands are parallel, and the two at the edges of the β-sheet are antiparallel. The mixed β-sheet is sandwiched between five α-helices, two of them flanking one side (α1–α2) and the rest flanking the other side (α3–α5) (figure 1*b,c*). This folding is very similar to that found in other macrodomains belonging to other kingdoms of life, as reflected by the low r.m.s.d. obtained compared with those deposited in the PDB database. However, some macrodomains show only a six-stranded mixed β-sheet, since they lack the β1 strand found in OiMacroD, as it has been described in *E. coli* YmdB (EcYmdb) [11], in some viral macrodomains (SARS-CoV and IBV nsP3 macrodomains, CHIKV and VEEV nsP3 macrodomains) [23–26] and in hMacroD1 [8] (electronic supplementary material, figure S3). Despite this, superimposition of OiMacroD with the bacterial homologues EcYmdB (1SPV) and the hypothetical protein TTHA0132 from *Thermus thermophilus* HB8 (2DX6) showed an r.m.s.d. of 0.98 Å (165 Cα atoms) and 1.33 Å (149 Cα atoms), respectively. Pairwise superimposition with members of other kingdoms, such as hMacroD1 (2X47) or Af1521 protein from the archaea *Archaeoglobus fulgidus* (2BFQ) [6], also provided very close values, with an r.m.s.d. of 1.27 Å (161 Cα atoms) and 1.62 Å (178 Cα atoms), respectively.

**Table 1.** Data collection and refinement statistics.

	MES	ADP-ribose	ADP
PDB code	5FUD	5L9K	5L9Q
*data*			
space group	*P*2_1_	*P*2_1_	*P*2_1_
cell dimensions			
*a*, *b*, *c* (Å)	47, 96.7, 54.4	47.4, 96.7, 54.4	47.4, 95.7, 54.7
*α*, *β*, *γ* (°)	90, 115.7, 90	90, 115.8, 90	90, 115.6, 90
resolution (Å)	1.90	1.77	1.75
*I*/*σ*_I_	6.3 (1.9)^a^	13.1(2.2)	9.9 (2.3)
*R*_sym_ (%)	9.8 (35.6)	6.6 (47.0)	6.7 (32.8)
*R*_meas_ (%)	12.0 (43.8)	7.7 (56.5)	8.6 (43.0)
completeness (%)	96.3 (93.8)	97.2 (84.1)	91.7 (63.0)
redundancy	2.9 (2.7)	3.6 (2.8)	2.2 (1.9)
*refinement*			
resolution (Å)	20–1.9	20–1.77	20–1.75
no. of reflections	31435	39295	37246
*R*_work_/*R*_free_ (%)	17.1/21.2	15.2/16.9	17.0/19.8
B-factors (Å2)			
protein	16.0	24.0	19.6
ligand	11.9	17.2	15.0
water	21.2	31.4	23.4
no. of atoms			
protein	2899	2921	2919
ligand/ion	35	72	108
water	218	245	186
r.m.s.d.			
bond lengths (Å)	0.009	0.010	0.009
bond angles (°)	1.30	1.72	1.56

	G37V (ADPr)	D40A	N30A
PDB code	5LAU	5LCC	5LBP
*data*			
space group	*I23*	*P*2_1_2_1_2_1_	*P*2_1_2_1_2_1_
cell dimensions			
*a*, *b*, *c* (Å)	109.6, 109.6, 109.6	53.7, 63.2, 120.4	43.8, 61.8, 68.8
*α*, *β*, *γ* (°)	90, 90, 90	90, 90, 90	90, 90, 90
resolution (Å)	1.35	2.0	1.75
*I*/*σ*_I_	22.2 (6.2)^a^	18.7 (3.9)	10.4 (3.6)
*R*_sym_ (%)	7.0 (40.4)	4.5 (36.3)	17.0 (61.7)
*R*_meas_ (%)	7.3 (42.2)	5.0 (40.2)	18.8 (69.0)
completeness (%)	100 (100)	90.8 (90.8)	99.1 (97.6)
redundancy	12.2 (12.0)	5.1 (5.4)	6.3 (5.4)
*refinement*			
resolution (Å)	40–1.35	15–2.0	19.8–1.76
no. of reflections	45534	24291	18654
*R*_work_/*R*_free_ (%)	15.4/18.2	18.5/23.1	20.5/23.7
*B*-factors (Å2)			
protein	18.4	42.8	15.4
ligand	24.2	—	—
water	28.2	44.3	24.9
no. of atoms			
protein	1439	2892	3122
ligand/ion	48	—	15
water	172	152	213
r.m.s.d.			
bond lengths (Å)	0.010	0.010	0.009
bond angles (°)	1.69	1.42	1.22

^a^Values in parentheses are data for the highest-resolution shell.

**Figure 2.**
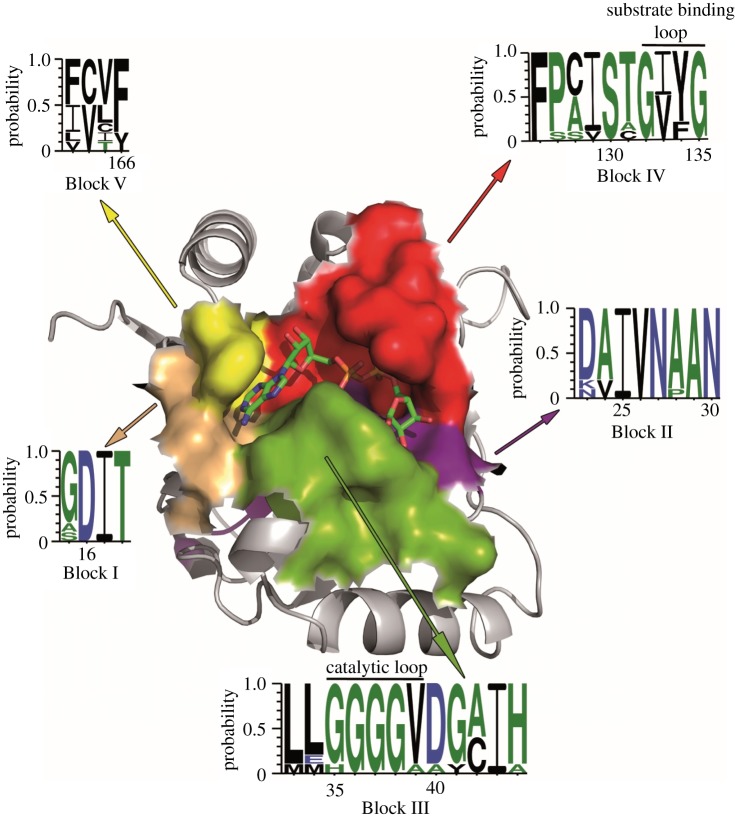
Surface representation of OiMacroD with bound ADPr. Conserved blocks forming the binding site of the protein are coloured. The consensus sequence was obtained as described in Material and methods. The β6–α4 and β3–α1 loops, previously named as *loop 1* or diphosphate-binding loop, and *loop 2* or catalytic loop, respectively, are also shown in the figure [7,9].

On its surface, the OiMacroD structure shows the active site in an L-shaped cleft (figure 1*b–e*), which contains most of the conserved blocks (I–V) (figure 2; electronic supplementary material, figure S3), with a volume higher than 300 Å^3^. The crevice is located over strands β3 (Block II), β6 (Block IV) and β7 (Block V), and is surrounded by loops connecting β2–β3 (Block I), β3–α1 (Block III) and β6–α4 (Block IV) (figures 1*b,c* and 2; electronic supplementary material, figure S3). Electron-density (ED) maps of native crystals soaked with ADPr (OiMacroD-ADPr) or ADP (OiMacroD-ADP) show a large mass of ED that matches an ADPr or ADP molecule bound to the enzyme and follows the same pattern found in other MacroD-like structures (figure 1*d*) [7]. The adenine moiety is stacked in a hydrophobic pocket with the highly conserved residues F166, I17 and V39 (figure 1*d,e*). In addition, it is also strongly bound to residues D16 and I17 by main-chain interactions, which belong to the highly conserved sequence DIT in Block I (figures 1*e* and 2). This latter sequence is almost fully conserved, with just a few exceptions in viral macrodomains and in the binding macrodomains of hPARP14 and hPARP15 (electronic supplementary material, figure S3), where the third position in Block I is less conserved. By contrast, the proximal ribose, showing a 2′endo pucker conformation, does not maintain direct interaction with any residue of the protein, although it interacts with different water molecules through H-bonds (see below) (figure 1*e*).

The pyrophosphate moiety of ADPr exhibits a rich network of contacts, which are decisive for ADPr binding. This pyrophosphate moiety is surrounded by helix α1 and the P-loop-like β6–α4, previously named as *loop 2* or diphosphate-binding loop (figures 1*b,c* and 2) [7,9], with its positive dipoles oriented towards the phosphates, thus stabilizing the negative charges (figure 1*b,c,e*). Residues S130, G132, V133 and Y134, belonging to the P-loop-like motif (Block IV), and V39 (Block III) in helix α1, are directly involved in diphosphate binding (figures 1*e* and 2; electronic supplementary material, figure S3). Finally, the distal ribose fitted in a cavity surrounded by the glycine-rich loop β3-α1 (Block III, G35–V39 in OiMacroD), also termed *loop 1* or catalytic loop (figures 1*b,c* and 2) [7,9], and residues V133 and Y134 from the P-loop-like motif (Block IV). Residue G37 (loop β3–α1) anchors the distal ribose 1″-OH group, whereas the 2″-OH group is bound to residues N30 (loop β3–α1) and D40 (helix α1) (figure 1*e*).

**Figure 3.**
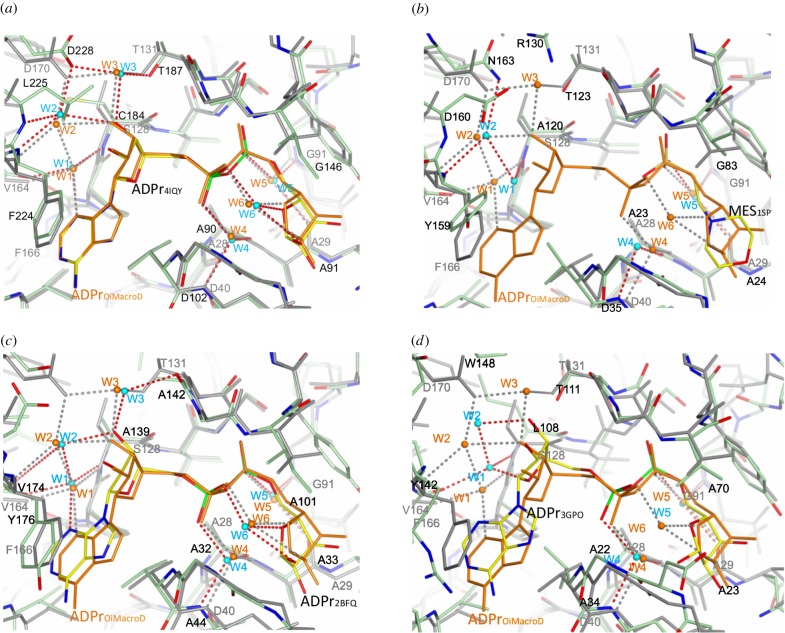
Structural comparison of OiMacroD with different macrodomains. OiMacroD-ADPr structure (grey) is superimposed with the crystal structure (green) of (*a*) human MacroD2 (PDB 4IQY), (*b*) EcYmdb macrodomain (PDB 1SPV), (*c*) hypothetical protein Af1521 from *A. fulgidus* (PDB 2BFQ) and (*d*) Chikungunya virus macrodomain (PDB 3GPO). The ADPr molecule bound to OiMacroD is shown (orange) superposed to the ligand present in each homologue (yellow). Water molecules are represented as orange (OiMacroD) or cyan (superposed macrodomain) spheres. The interactions established by each structural water molecule are represented as dashed lines in grey (OiMacroD) or red (superposed macrodomain).

Of note is the finding of a set of five well-ordered water molecules (W1–W5) fixed in the active centre of OiMacroD. The presence of these water molecules represents a constant, previously undescribed element in the macrodomain binding site of Bacteria, Archaea and Eukarya, even in the absence of ligands. These water molecules are placed in key positions, establishing interactions with the different portions of the ADP moiety and forming a highly conserved network of interactions (figure 1*e*). The high resolution of some of the structures resolved and the relatively low B-factor value found for these waters, which denotes the absence of mobility, supports their structural role (electronic supplementary material, table S1). A precise H-bond network for adenosine moiety binding was found in both the presence and absence of ligand, consisting of three fixed conserved water molecules (W1–W3). W1 anchors the adenine N3 atom in the correct place, and is fixed to the protein by means of main-chain interactions with residues V164 and S128 (figure 1*e*). Interestingly, two water molecules (W2–W3), fixed by residues F166, D170 and T131, are accurately positioned to bind the proximal ribose 3′-OH. These three water molecules are also found in bacteria, archaea or human macrodomain structures (1SPV, 2DX6, 2X47, 2BFQ and 4IQY) (figure 3), and even in non-catalytic domains, with minimal differences (3Q6Z, 3Q71, 3IID). Therefore, although the proximal ribose is apparently not bound to the protein, this water network precisely anchors the 3′-OH atom in the correct place.

Complementary to W1–W3, two additional structural water molecules (W4–W5) were found in OiMacroD, where they are involved in pyrophosphate binding. W4 is anchored to the protein by main-chain interactions with residues D40 (helix α1) and A28 (strand β3), helping to bind the ADPr α-phosphate (Pα), whereas W5 anchors the β-phosphate (Pβ) by establishing main-chain contacts with the conserved residues A29 (strand β3) and G91 (strand β5) (figure 1*e*). These two latter structural waters are also present in bacteria, human, archaea and viral macrodomains (1SPV, 2X47, 4IQY, 2BFQ, 3Q6Z, 3GQO and 3GPO) (figure 3). Interestingly, although W4 is present in all the structures analysed, W5 is replaced by the side chain of residue S302 (A28 in OiMacroD) in the second binding macrodomain of hPARP15 (3V2B) (electronic supplementary material, figures S3 and S4), mimicking the same interaction established by W5 with the Pβ. The conservation of these interactions with W4–W5 in different groups of macrodomains points to its important role in ADPr stabilization (figure 3). Consequently, these two water molecules play a role in pyrophosphate binding, allowing the substrate to adopt a suitable conformation for catalysis.

Both sets of structural water molecules are also present in the catalytic domain of the hPARG, except W2, which is substituted by residue N869 (PDB 4B1H) [27], demonstrating that this set of water molecules is not only characteristic of MacroD-like macrodomains but also of macrodomain-related folds. Furthermore, these structural water molecules (W1–W5) provide roughly 40% of all the interactions established with the ligand, representing a key organizing element of the macrodomain-binding site, with an important role in substrate binding.

### 2.3. Loop β6–α4 is essential for pyrophosphate recognition and distal ribose orientation

Although a substantial amount of work has been carried out in the study of macrodomains, few papers have reported the presence of conformational changes induced by substrate binding. Such changes have been established by comparing structures from different MacroD-like macrodomains [9], or through the study of OARD1 macrodomain [14], which represents a phylogenetically distinct variant (electronic supplementary material, figure S2). However, these conformational movements were clearly observed in OiMacroD. Although superimposition of the OiMacroD structures revealed the lack of important overall conformational changes (r.m.s.d. 0.3–0.6 Å for 186 Cα atoms), this comparison revealed the presence of key changes around the active site that favoured ADPr binding. Such is the case with residue F166 in OiMacroD, which undergoes a rotation that favours a π-stacking interaction with the adenine ring of ADPr, thus facilitating its binding (figure 1*e*; electronic supplementary material, figure S5) [7,8].

**Figure 4.**
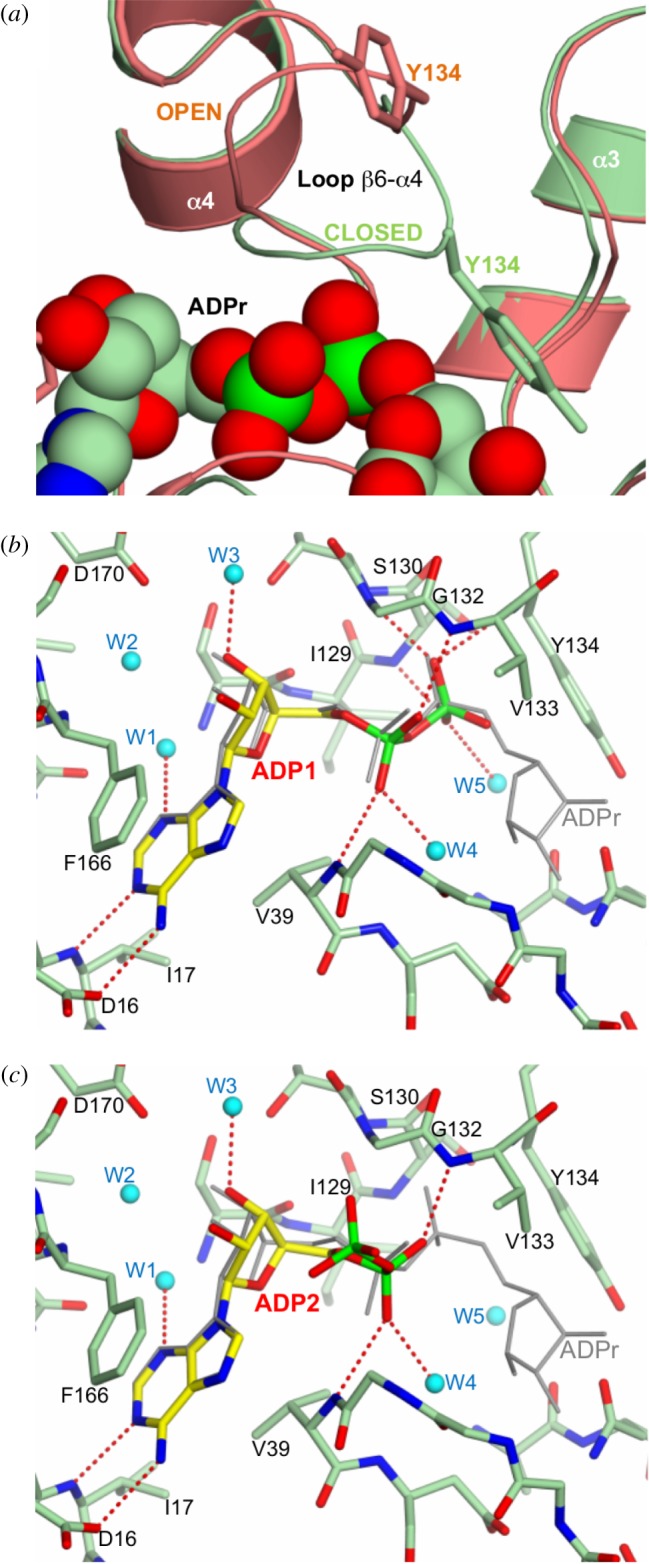
Substrate-induced changes during ADPr binding. (*a*) Superimposition of the OiMacroD-ADPr (green) and D40A structures (salmon), highlighting the closure of the β6–α4 loop induced upon ADPr binding with the concomitant movement of Y134. (*b*,*c*) Interactions established by the two alternative conformations of the ADP molecule (Cα in yellow) observed in the OiMacroD-ADP structure. The ADPr (grey) found in OiMacroD-ADPr structure was superimposed. Water molecules are represented as spheres (cyan).

In addition, two different conformations (open and closed) are evident in the β6–α4 loop (residues 131–135) [9,14]. The closed form appears when ADP or ADPr is bound, covering and coordinating the pyrophosphate moiety (figure 4*a*, green). A rigid body outward movement of 5.5 Å (hinge in residues T131 and G135) takes place when the active site is empty, yielding a fully open conformation, as observed in the D40A mutant (figure 4*a*, salmon). Interestingly, both alternative conformations of the β6–α4 loop are visible in the same subunit of the N30A mutant, representing a snapshot of the complete conformational cycle of this loop (electronic supplementary material, figure S6). Thus, the β6–α4 loop acts as a clamp over the pyrophosphate, occluding this charged moiety inside the protein.

One consequence of this induced-fit movement was the large displacement (approx. 15 Å) of residue Y134, which is incorporated in the active site when substrate is present (figure 4*a*, green). However, the latter residue is replaced by residue D125 in OARD1 (PDB 2L8R, 4J5S) [5,14], which is immediately opposite K84. Both amino acids form the catalytic dyad of OARD1 and cover the equivalent space of residue Y134. This fact permits the distal ribose to adopt conformations not possible in MacroD-like macrodomains [5]. This is another important difference between MacroD-like and OARD-like macrodomains to add to those related to the different phylogenetic origins [7], low sequence homology (only 16%) or structural differences (r.m.s.d. 2.32 Å for 128 Cα atoms). The importance of this Y134 has been shown previously, when mutation of the equivalent residue Y126A in EcYmdB macrodomain (a macroD-like macrodomain) was carried out [11]. This mutation completely abolished activity, thus indicating that the loss of the phenyl ring to stabilize OAADPr distal ribose may make the substrate too flexible to be hydrolysed [11]. The importance of this phenyl ring to stabilize substrate binding has also been observed in the hepatitis E virus macrodomain fused with a helicase domain (Macro-Hel), where the equivalent Y125A mutant yielded an inactive enzyme, whereas Y125F showed comparable de-MARylation activity to the WT [28]. Thus, this closure of the β6–α4 loop associated with ADPr binding in MacroD-like macrodomains, together with the positioning of residue Y134 over the distal ribose, is essential for the proper orientation and stabilization of the substrate for catalysis, confirming previously described results [11,28].

However, an exception to this rule has recently been reported in the *Trypanosoma brucei* macrodomain, which shows a preformed ADPr site [12]. In this case, when the apo-form is compared with the ADPr bound form, the P-loop-like does not experiment any conformational change but always displays the closed conformation (PDBs 5FSU, 5FSY). Curiously, the homologous structure in *Trypanosoma cruzi* (64% homology) shows a similar conformational open/closed movement of the P-loop-like to that found in OiMacroD. Taking into account that residues in the P-loop-like and the nearby area are almost identical in both *Trypanosoma* proteins, this conformational difference seems puzzling. The only difference in sequence near the P-loop-like corresponds to V178 in *T. brucei,* which is found to be an isoleucine in *T. cruzi* macrodomain (I171 in 5FSZ), hMacroD1 (I230 in 2X47) and OiMacroD (I93), three examples of macrodomains requiring a P-loop-like conformational change for catalysis. Surprisingly, both trypanosomatid macrodomains in the corresponding apo forms (5FSV and 5FSZ, respectively) only presented the active conformation of the residue equivalent to F166 in OiMacroD, which is involved in adenine binding (F248 and F241 in *T. brucei* and *T. cruzi*, respectively). This active conformation might be explained by CH/π interactions between a proline residue (P108 and P101 in *T. brucei* and *T. cruzi*, respectively) and its corresponding phenylalanine residues [29], which lock it in the active form. This proline residue is an aspartate in OiMacroD and hMacroD1 (D16 and D160, respectively), a residue unable to establish this sort of interaction.

**Figure 5.**
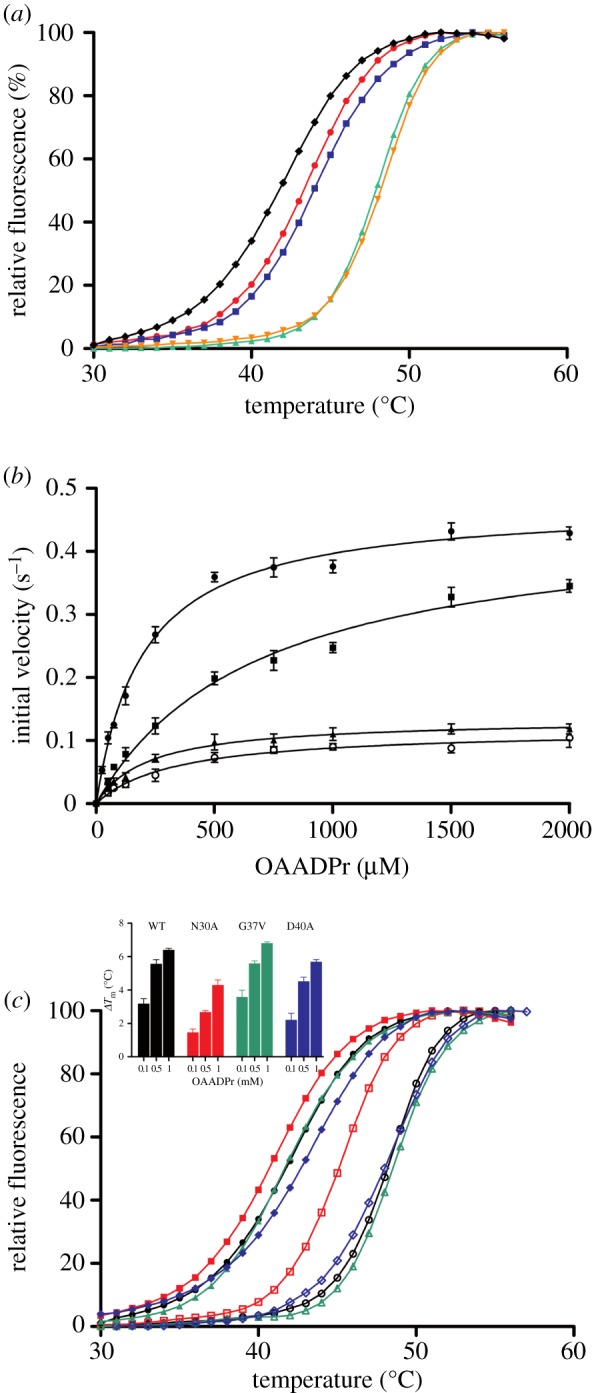
Deacetylation of OAADPr and thermostability of OiMacroD and its mutants. (*a*) Melting temperature curves of purified OiMacroD WT with different ligands. Curves were obtained in the presence of the fluorescent probe SYPRO Orange in 50 mM sodium phosphate pH 7.0: no ligand (diamonds), 1 mM AMP (circles), 1 mM ADP (squares), 1 mM ADPr (triangles) or 1 mM OAADPr (inverted orange triangles). (*b*) Effect of substrate concentration on OiMacroD activity (filled circles) and its N30A (squares), G37V (triangles) and D40A (open circles) mutants. The error bars represent standard deviations calculated from the initial velocities at each substrate concentration from three separate experiments. (*c*) Melting temperature curves of purified OiMacroD and its mutants. Curves were obtained in 50 mM sodium phosphate pH 7.0 in the absence of ligand for WT (closed black circles), N30A (closed red squares), G37V (closed green triangles) and D40A (closed blue diamonds), or in the presence of 1 mM OAADPr for WT (open black circles), N30A (open red squares), G37V (open green triangles) and D40A (open blue diamonds). Inset: effect of OAADPr binding in OiMacroD and its mutants on melting temperature (*T*_m_) at three different substrate concentrations. The increase in *T*_m_ (Δ*T*_m_) was calculated by subtracting the *T*_m_ values obtained in the absence of ligand from those obtained in its presence.

The crystal structure of OiMacroD-ADP provides new details about both the mode of ADP binding and the role of the β6–α4 loop in this process, in addition to the data shown in the archaeal Af1521 macrodomain by Karras *et al*. [6]. They determined a 45-fold reduction in affinity for ADP compared with ADPr, and also described that ADP binds essentially as found in the ADPr complex. Interestingly, the OiMacroD-ADP complex showed two alternative conformations of the bound ADP molecule, in contrast to the single one found in Af1521 (figure 4*b,c*; electronic supplementary material, figure S7) [6]. In the first conformation, the ADP fitted roughly in the same way as the ADPr in OiMacroD and the ADP in Af1521 (figure 4*b*). However, a detailed view of the pyrophosphate showed that it remains slightly distorted. In this case, the β6–α4 loop was completely closed, as expected, over the Pα and Pβ, but lost some of the interactions established with the pyrophosphate, especially with residues V133 and Y134 (figure 4*b*). In the second conformation, the ED map revealed the ADP molecule to be bound to the protein only through the interactions with the adenosine and Pα moieties, whereas the Pβ is projected from the active site, remaining exposed to the solvent (figure 4*c*). In this alternative conformation, the Pβ lost the rich network of interactions characteristic of the ADPr complex, essentially presenting a snapshot of how AMP could bind to the protein. Surprisingly, in this second conformation, the β6–α4 loop remained closed and fully packed, due to a main-chain H-bond between the Pα and residue V133. Thermal shift assays carried out with OiMacroD show that AMP (*T*_m_ *=* 43.3 ± 0.1°C) protects the enzyme from thermal inactivation in a similar way to ADP (*T*_m_ *=* 44.0 ± 0.2°C), although to a lesser extent than ADPr (*T*_m_ *=* 47.8 ± 0.1°C) or OAADPr (*T*_m_ *=* 48.2 ± 0.1°C), as shown by the increase in its melting temperature (Δ*T*_m_) in the presence of AMP compared with that obtained in the absence of ligands (*T*_m_ *=* 41.7 ± 0.2°C) (figure 5*a*). This result indicates that AMP binds to OiMacroD. In fact, AMP protects the enzyme similarly to ADP, which suggests that both are able to bind to OiMacroD with similar strength (figure 5*a*). Taking these results into account, and the fact that no binding of adenosine alone was detected in Af1521 [6], AMP seems to be the smallest portion of the ADPr molecule that can bind to macrodomains, and is specifically recognized by loop β6–α4. This fact also highlights the essential role of the interaction between the Pα and V133 for AMP binding, which acts as a trigger to induce β6–α4 loop closure. Thus, the β6–α4 loop seems to be able to recognize the AMP moiety even when the Pβ is absent.

Collectively, these results illustrate the dual role in substrate binding of the β6–α4 loop in most MacroD-like macrodomains. The first is related to binding of the pyrophosphate to the protein, which triggers the closure of the loop through an induced fit mechanism, and the second is related to the stacking of the distal ribose in the correct place and properly oriented for catalysis by attracting residue Y134, as a consequence of the pyrophosphate-induced closure. In other words, the presence and proper orientation of the distal ribose is necessary for the correct fitting of the pyrophosphate, otherwise its binding to the β6–α4 loop will be distorted.

### 2.4. Mutant D40A reveals an additional structural role for residue D40

The proposed catalytic mechanism for MacroD-like proteins requires the presence of three conserved residues: an asparagine (loop β3–α1), an aspartate (helix α1) and a tyrosine (loop β6–α4) (electronic supplementary material, figure S3) [8,11]. The asparagine coordinates the nucleophilic water [15], the tyrosine stabilizes the orientation of the distal ribose [11] and the aspartate activates the catalytic water molecule. This latter residue acts as a general base, deprotonating the catalytic water to attack the carbonyl group with the concomitant hydrolysis of the acetyl group [8,11]. Additionally, an asparagine (β3) and a histidine (α1) assist in the catalysis by coordinating the aspartate, thus contributing to deprotonation of the catalytic water [8]. The above residues correspond to N30, D40, Y134, N27 and H44 in OiMacroD, respectively.

To gain insight into the structural and catalytic role of residues N30 and D40, the corresponding alanine mutants were constructed, instead of more conservative mutants, with the aim of completely abolishing the catalytic activity. Surprisingly, these mutants were active towards OAADPr (figure 5*b*). However, when their kinetic parameters were calculated (table 2), a significant reduction (72–86%) in catalytic efficiency was found compared with the WT value (2412 M^−1^ s^−1^). This catalytic efficiency of OiMacroD is similar to that described for bacterial catalytic macrodomains EcYmdB (3042 M^−1^ s^−1^) [11] and SaV0325 (1835 M^−1^ s^−1^) [8], but 4.5- and 2.1-fold higher than those of hMacroD1 (528 M^−1^ s^−1^) [8] and hMacroD2 (1121 M^−1^ s^−1^) [8], respectively.

**Table 2.** Kinetic parameters and melting temperatures for OiMacroD and its mutants.

OiMacroD	*K*_m_, µM	*k*_cat_, s^−1^	*k*_cat_/*K*_m_, M^−1^ s^−1^	*T*_m_ at pH 7.0, °C	*T*_m_ in OAADPr 1 mM, °C
WT	199 ± 23	0.48 ± 0.03	2412	41.8 ± 0.2	48.2 ± 0.1
N30A	618 ± 95	0.41 ± 0.03	663	40.8 ± 0.3	45.1 ± 0.3
G37V	218 ± 32	0.13 ± 0.01	596	41.7 ± 0.1	48.5 ± 0.1
D40A	332 ± 48	0.11 ± 0.01	331	42.8 ± 0.3	48.5 ± 0.2

The 72% reduction in catalytic efficiency found in N30A mutant was not due to overall changes in the structure, whether crystallized or in solution, since its *T*_m_ was close to that of WT in 50 mM sodium phosphate pH 7.0 (figure 5*c*; table 2). The main difference found was the threefold increase in the *K*_m_ value, with practically no effect on its *k*_cat_. This decrease in N30A affinity was also detected in the thermal shift assay (figure 5*c* inset; table 2), where slightly lower Δ*T*_m_ values were observed in the presence of OAADPr at all the concentrations used compared with those obtained with WT (figure 5*c* inset; table 2). However, the 86% reduction in catalytic efficiency found in D40A mutant was basically due to a 4.4-fold decrease in *k*_cat_, accompanied by a slight increment in the *K*_m_ value, which was also observed in its Δ*T*_m_ in the presence of OAADPr compared with the WT (figure 5*c* inset; table 2). This increase in *K*_m_ was also described when this aspartate was mutated in EcYmdb (D35A) [11].

**Figure 6.**
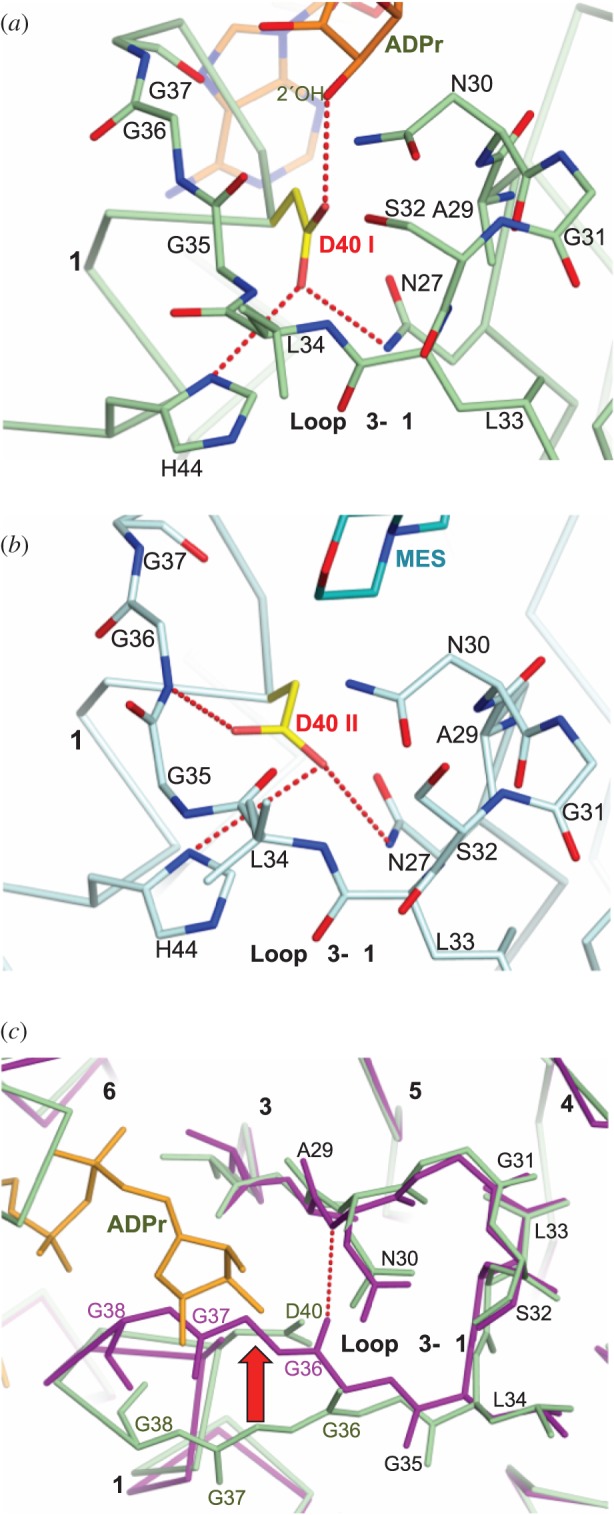
Structural role of residue D40. (*a,b*) Interactions established by the D40 rotamers (yellow). (*a*) Residues surrounding rotamer D40 I (OiMacroD-ADPr complex) and (*b*) rotamer D40 II (OiMacroD-MES complex). (*c*) Displacement of β3–α1 loop (red arrow) in the D40A mutant (purple) compared with that of the OiMacroD-ADPr complex (green). The ADPr molecule is coloured orange.

To gain insight into the role of this residue, the D40A mutant structure was analysed and compared with the other OiMacroD complexes, allowing us to identify the presence of two different D40 rotamers. The first and well-known rotamer (rotamer I) interacted with the distal ribose 2″-OH atom, playing a role in substrate binding and catalysis (figure 6*a*). Rotamer I was also found in EcYmdb (5CB3) and hMacroD2 (4IQY). Nevertheless, a new second rotamer (rotamer II) was observed in the OiMacroD-MES, N30A mutant and EcYmdb apo-form (1SPV) structures, where it was seen to interact with the β3-α1 loop through the amide N atom of residue G36 (figure 6*b*). This interaction was favoured by the reorganization of residues L34 and G35 belonging to the loop. Rotamer II appears when no ADPr is present due to the absence of the 2″-OH group of the distal ribose. Interestingly, in D40A mutant, the absence of the H-bond between D40 and G36 structure caused a major displacement of the β3–α1 loop of about 4 Å towards the distal ribose, occluding the distal ribose binding site (figure 6*c*). This conformation was stabilized by a main-chain interaction between residues G36 and N30, which is made possible by the absence of the D40 side-chain, allowing the β3–α1 loop to occupy part of the remaining free space. Soaking or co-crystallization of this mutant in the presence of ADPr yielded no complex. Hence, this study suggests a novel role for residue D40 in the stabilization and organization of the β3–α1 loop, maintaining the proper conformation of the distal ribose site for OAADPr binding. The displacement of the β3–α1 loop could also explain the lower Δ*T*_m_ upon OAADPr binding at different concentrations (figure 5*c* inset), and the slight increase in *K*_m_ (table 2) compared with WT. Collectively, these results suggest a dual role for residue D40, a catalytic role as the main activator of the catalytic water and a novel structural function in the organization of the β3–α1 loop, which is essential for maintaining the proper architecture of the binding site for ADPr and related molecules, as has been previously described [9,26,30].

### 2.5. Mutant G37V provides evidence for the participation of a substrate-coordinated water molecule in catalysis

The proposed catalytic mechanism of OARD1 macrodomain is based on the absence of OAADPr deacetylation when residue D125 belonging to the catalytic dyad is mutated [5,14]. However, the corresponding mutation in OiMacroD (D40A), or even that in N30A, substantially reduces the catalytic efficiency, as mentioned above, but without completely abolishing the activity (table 2). The inability of these mutations to eliminate the activity has also been observed in other MacroD-like macrodomains [9,11]. In addition, there are two MacroD-like macrodomains (Af1521 and TTHA0132) where this proposed catalytic aspartate is naturally replaced by alanine (A44 or A34, respectively), and at least one of them (Af1521) has been demonstrated to be active [9]. These findings suggest the possible existence of a different pool of catalytic residues or a different catalytic mechanism in MacroD-like macrodomains.

**Figure 7.**
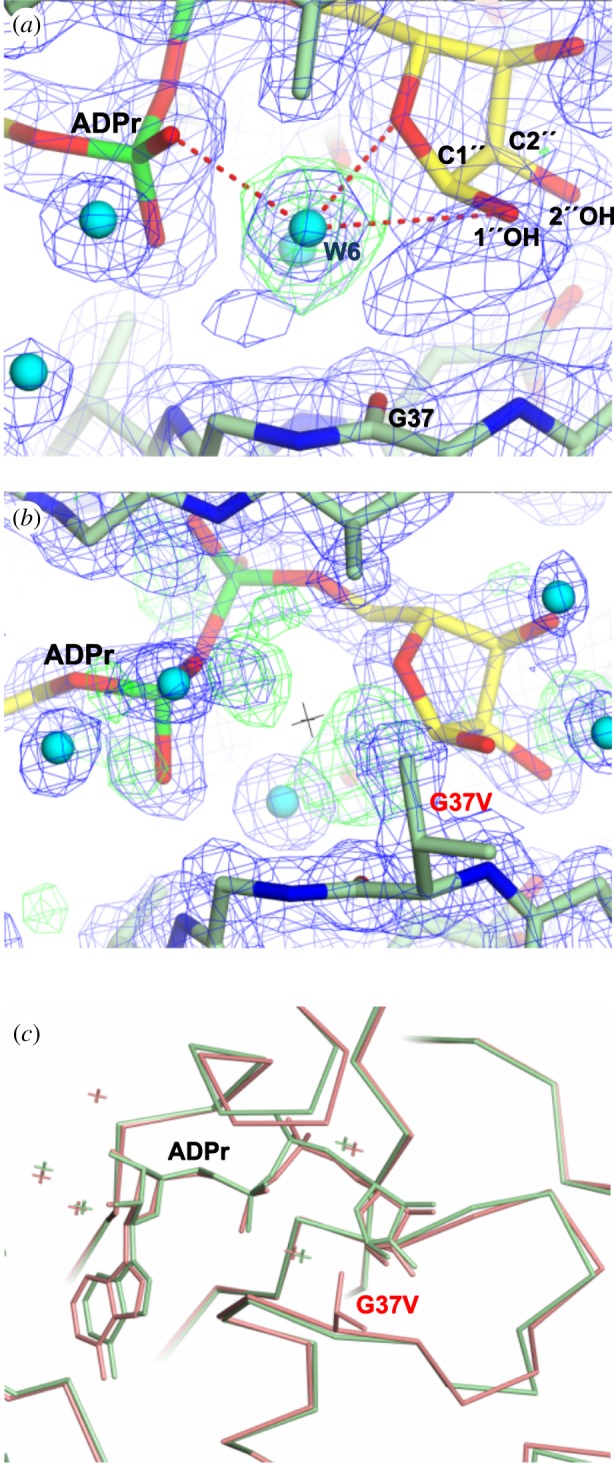
Participation of a substrate-coordinated water molecule in catalysis. (*a*) ADPr coordination of the proposed substrate-coordinated water (W6) in OiMacroD-ADPr structure. The *2Fo−Fc* ED map (1.0 σ; blue) is shown for all the elements, while the *Fo−Fc* omit ED map (2.5 σ; green) is only shown around W6. (*b*) The absence of the substrate-coordinated water in the G37V mutant. The black cross in the centre of the figure indicates the position of W6 in OiMacroD-ADPr. *2Fo−Fc* (1.0σ; blue) and *Fo−Fc* (2.5σ; green) ED maps highlighting the extra positive ED around the pyrophosphate and distal ribose. (*c*) Superimposition of the OiMacroD-ADPr (green) and mutant G37V (red) structures illustrating the absence of structural changes.

In light of this, Jankevicius *et al*. [9] proposed an alternative substrate-assisted mechanism for hMacroD2, in which a water molecule coordinated by the substrate and activated by the Pα is responsible for the nucleophilic attack on the ribose C1″ atom [9]. However, G100E, I189R and Y190N mutants (G38, V133 and Y134 in OiMacroD, respectively), which were designed in the above study to test this hypothesis, did not manage to displace this substrate-coordinated water molecule without hampering ADPr binding [9]. In addition, there is controversy over whether the low established pKa of Pα (approx. 2) is sufficient to activate the water molecule or not [31]. Since this water molecule is also present in the OiMacroD-ADPr structure (W6) with a good B-factor value, where it interacts with the Pα and the distal ribose through the 1″-OH and ether O atom (figure 7*a*; electronic supplementary material, table S1), an OiMacroD G37V mutant was designed to study its role.

This novel G37V mutant showed a drop in its catalytic efficiency that did not substantially affect substrate affinity or the corresponding melting temperature in the presence or absence of OAADPr (figure 5*c* inset; table 2). The structural analysis of G37V, which contains an ADPr molecule bound, revealed a lack of ED for W6 because the substrate-coordinated water molecule is absent (figure 7*b*), unlike in the OiMacroD-ADPr complex (figure 7*a*). This absence does not hamper ADPr binding or cause any perturbation in the protein structure (figure 7*c*). As expected, the valine side chain is located close enough to the water molecule position to disturb its coordination with the ADPr molecule (figure 7*b*). However, this slight perturbation reduced the *k*_cat_ of the G37V mutant fourfold, to reach a value similar to that found in D40A (table 2), pointing to a possible catalytic role. Interestingly, although the *K*_m_ remained unaffected, some extra positive ED is seen to surround the pyrophosphate and especially the distal ribose, reflecting some uncertainty in the position of the atoms (figure 7*b*). This result contrasts with the ED found in the OiMacroD-ADPr complex, where the pyrophosphate and distal ribose are correctly positioned and perfectly anchored to the protein (figure 7*a*). As a consequence of the disorder found in the distal ribose of the G37V mutant, both D40 rotamers were present, supporting the role of this residue in the structural organization of the β3–α1 loop. Hence, W6 seems to be important for the substrate to acquire the correct conformation, favouring the proper alignment of the reactive groups and leading to OAADPr deacetylation.

**Figure 8.**
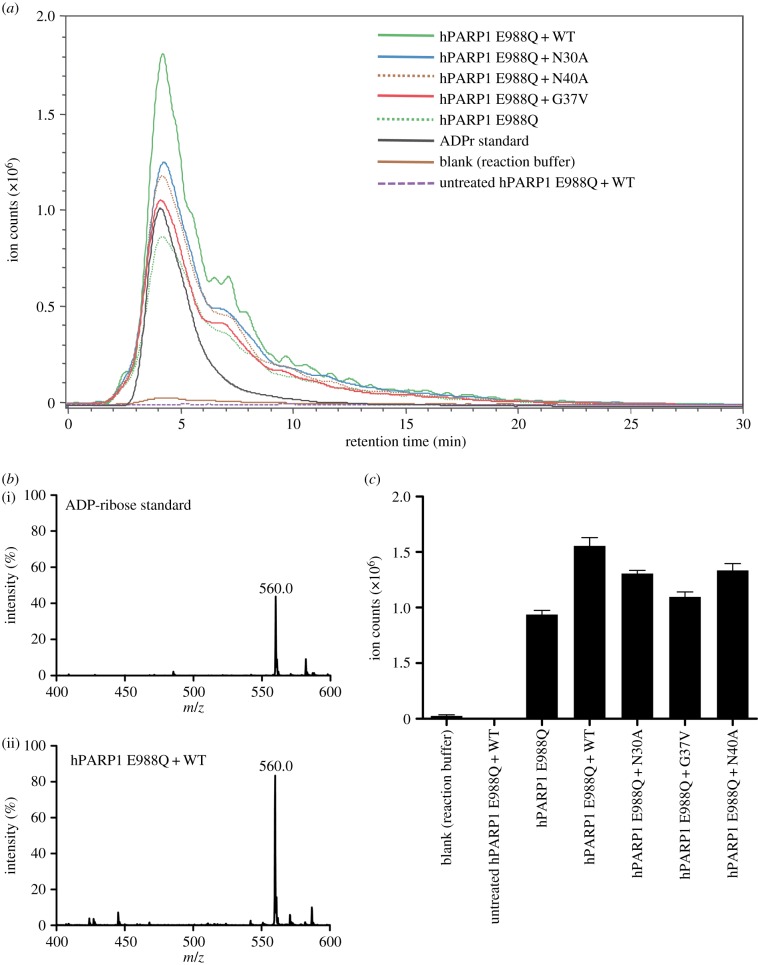
OiMacroD de-MARylation activity. (*a*) Extracted ion chromatograms of the monoisotopic mass of the ADP-ribose [M + H]^+^ adduct (560 *m/z*) for the ADP de-ribosylation reactions of OiMacroD WT and its mutants. (*b*) MS analysis of ADPr standard (i) and hPARP1 E988Q-OiMacroD WT reaction (ii). The 560.0 *m/z* corresponds to ADPr. (*c*) Ion abundance of mass 560.0 for OiMacroD WT and mutants in de-MARylation reactions. Error bars represent the standard deviation of three replicate runs. hPARP1 E988Q represents the mono-ADP-ribosylated protein, whereas untreated hPARP1 E988Q means the unmodified protein. The blank is the reaction buffer used to produce MARylated hPARP1 E988Q. OiMacroD WT and its mutants are designed as WT, N30A, G37V and D40A, respectively.

Recent studies have proved that MacroD1/D2, TARG1, protein Af1521 and viral macrodomains can reverse mono-ADP-ribosylation using mono-ADP-ribosylated proteins as substrate [5,9,10,28]. To test whether OiMacroD is capable of releasing mono-ADP-ribose (MAR) from modified proteins or not, mono-ADP-ribosylated hPARP1 E998Q mutant, an accepted model of mono(ADP-ribosyl)ated PARP1 substrate [31,32], was co-incubated with OiMacroD WT or its corresponding mutants. When analysed by HPLC-ESI-MS/MS, the released ADPr showed an elution peak that in all cases coincided with the retention time and molecular mass (560 *m/z*) of the ADPr standard (figure 8*a,b*). These results indicate that OiMacroD is an enzyme able to remove mono-ADPr units from MARylated hPARP1 E998Q. Interestingly, the OiMacroD mutants also showed reduced de-ribosylation activity compared with the WT (figure 8*a*,*c*). In particular, mutant G37V revealed a significant reduction in ADPr release, which supports the role of W6 not just in OAADPr deacetylation, but also in the removal of ADPr from MARylated proteins.

Collectively, these results support the hypothesis that the substrate-coordinated water molecule (W6) plays a role in catalysis since no other set of catalytic residues has been found to date. This water molecule has been identified as being important for the catalytic activity of hMacroD2 and viral macrodomains [9,28], and seems to be a common trait in the catalytic mechanism of MacroD-like macrodomains. However, in OAADPr hydrolysis, the C2″ atom of the distal ribose is situated 4.7 Å from this water molecule, a distance that would hinder the nucleophilic attack on the acetyl group of 2′-OAADPr. Since the nearest hydroxyl to W6 is 1″-OH, when activated by Pα, it could carry out the nucleophilic attack on the distal ribose C1″ atom and cause OAADPr deacetylation [9]. This hypothesis requires the presence of the 1′-OAADPr isomer to maintain the acetyl group at a proper distance for the nucleophilic attack to occur. This isomer has been identified at neutral and basic pH values in rapid equilibrium with the 2′-OAADPr and 3′-OAADPr isomers [33]. Consequently, the nucleophilic attack performed by the activated water on the C1″ atom of the 1′-OAADPr isomer may explain the enzyme activity on OAADPr and mono-ADPr-ribosylated hPARP1 E998Q when the supposed catalytic residues are mutated. In fact, the Af1521 macrodomain, which naturally lacks the catalytic aspartate, exhibits ADP-ribose-1″ phosphatase activity, generating ADPr and inorganic phosphate as products [6], supporting this catalytic mechanism. Also, this seems to be the case for the catalytic mechanism of the viral macrodomains from *Coronaviridae*, *Togaviridae* and *Hepeviridae* families [28]. Whatever the case may be, the presence of this substrate-coordinated water molecule seems to play an important role in the OiMacroD activity and probably in other MacroD-like macrodomains, by setting up the right conformation of the substrate for OAADPr deacetylation or, alternatively, for de-mono-ADP-ribosylation.

## 3. Concluding remarks

This study advances our understanding of substrate binding and catalysis of macrodomains in a double approximation: the biochemical characterization of three catalytic site point mutants, and their structural characterization using X-ray crystallography together with three additional complexes bound to MES, ADPr and ADP. This study provides fresh evidence that the active site of macrodomains contains a set of five structural water molecules that play an important role in substrate binding. Three of these waters are devoted to adenosine binding, while the other two are dedicated to pyrophosphate binding. Another key element, involved not just in substrate binding but also in catalysis, is the β6–α4 loop. The substrate-induced rigid body closure of this element acts as a clamp over the pyrophosphate, occluding this charged moiety inside the protein, where it has two roles: to recognize the pyrophosphate through main-chain amide interactions and to drag residue Y134 to the active site, stacking the distal ribose in the right place with an orientation suitable for catalysis. In addition, D40A mutant revealed an additional structural role for this residue, apart from the catalytic one: maintaining the proper architecture of the OAADPr site through the organization of the β3–α1 loop. Besides, a comparison of the six crystal structures obtained in this study, especially the one corresponding to G37V mutant, supports the idea that both previously proposed catalytic mechanisms are possible. Under normal conditions (WT structure), the conserved aspartate acts as a base to deprotonate a nearby coordinated water molecule, facilitating the ester bond hydrolysis of the OAADPr. However, when this aspartate is absent, as occurs in Af1521 or in D40A mutant, they could carry out 1′- OAADPr deacetylation through a nucleophilic attack on the C1″ atom by activating a water molecule bound to OAADPr molecule. This fact could be favoured by the rapid equilibrium reached between the different isomers of the OAADPr. Furthermore, as in human, archaeal and viral macrodomains, bacterial macrodomains are able to reverse mono-ADP-ribosylation from target proteins. Finally, this substrate-coordinated water molecule also seems to be involved in the catalysis of OiMacroD, being indispensable for the adequate conformation of the OAADPr by fixing the Pα and distal ribose in their respective places, as revealed by the fall in the catalytic efficiency observed towards OAADPr and mono-ADP-ribosylated substrates in the G37V mutant.

## 4. Material and methods

### 4.1. Plasmid constructs, protein expression and purification

The OiMacroD gene (GenBank BAC14244.1) was amplified by PCR using the genomic DNA from *O. iheyensis* and a high-fidelity thermostable DNA polymerase (Pfu UltraII Fusion HS, Agilent). The respective forward and reverse oligonucleotides were 5′-CACTC*GCTAGC*ATGAAACATAATATAAATGATAATACG-3′ and 5′-GGC*CTCGAG*TTAGATTTTTTCTATTAAATACTTCAAT-3′, which contained restriction sites (in italics) for *Nhe*I (forward primer) and *Xho*I (reverse primer). The amplified PCR product (558 bp) was inserted into the corresponding restriction sites of a pET28a plasmid (Novagen), yielding the plasmid pET28a-OiMacroD, which encodes the protein carrying an N-terminal His_6_-tag. All OiMacroD point mutations (N30A, G37V, D40A) were created as previously described [20], using plasmid pET28a-OiMacroD as template, followed by sequencing of purified plasmid DNA (QIAprep spin miniprep kit, Qiagen) to confirm the desired mutation. OiMacroD was expressed into *E. coli* Rosetta 2 (DE3) cells (Novagen) by growing them in Terrific Broth medium supplemented with 50 µg ml^−1^ kanamycin and 12.5 µg ml^−1^ chloramphenicol, and induced with 1 mM isopropyl-β-thiogalactopyranoside for 16 h at 25°C. Cell pellets were harvested by centrifugation at 5000*g*, resuspended in lysis buffer (50 mM sodium phosphate pH 8.0) and disrupted in a Bead Beater type homogenizer (BioSpec). Lysates were cleared by ultracentrifugation (100 000*g*) followed by Ni^2+^-chelating affinity chromatography (ÄKTA Prime Plus, GE Lifesciences) at 4°C using increasing concentrations of imidazole (40–250 mM) in 50 mM sodium phosphate buffer pH 7.3 with 500 mM NaCl. OiMacroD fractions were pooled, desalted, concentrated by dialysis and applied onto a Superdex 200 HiLoad 16/600 gel filtration column (GE Lifesciences) at 4°C in 50 mM sodium phosphate buffer pH 7.3 with 150 mM NaCl. Superdex fractions containing the pure protein (monitored by SDS–PAGE) were stored at −20°C with 10% glycerol. Mass spectrometry (HPLC/ESI/ion trap system) was used to confirm the molecular mass of the protein (22.6 kDa).

### 4.2. *O*-acetyl-ADP-ribose synthesis and purification

OAADPr was enzymatically synthesized using yeast deacetylase HST2 (yHST2), which was kindly provided by J. M. Pascal (Thomas Jefferson University, PA, USA). The reaction was carried out at 37°C in 50 mM Tris–HCl pH 7.3 containing 20 µM yHST2, 5 µM OiNIC [20], 350 µM NAD^+^ and 525 µM acetylated peptide [8]. OAADPr was purified by HPLC through a 4.6 × 250 mm C_18_ 5 µm Gemini column (Phenomenex) using MilliQ water with 0.015% TFA (solvent A) and acetonitrile with 0.02% TFA (solvent B) as mobile phases. The HPLC method consisted of a linear gradient from 0% to 8% solvent B for 12 min (1 ml min^−1^). Fractions were pooled and characterized by mass spectrometry to confirm the presence of the pure compound (electronic supplementary material, figure S8), which was a mixture of the 2′ and 3′ isomers of OAADPr, as previously described [33]. Pure OAADPr was lyophilized, dissolved in MilliQ water, flash-frozen in liquid nitrogen and stored at −80°C.

### 4.3. Activity assay

OAADPr deacetylation activity was assayed by monitoring the OAADPr consumption at 25°C in a reaction volume of 300 µl containing 50 mM sodium phosphate buffer pH 7.3, 0.1 µM OiMacroD and 500 µM OAADPr. A control assay without OiMacroD was also carried out in the same conditions to determine OAADPr autohydrolysis. *K*_m_ values were determined varying OAADPr concentrations (0–2 mM). Aliquots were taken at different times and the reaction was stopped with 1% TFA (v/v). After centrifugation, supernatants were injected into a 4.6 × 250 mm C_18_ column and OAADPr consumption was analysed by HPLC (1100 series Agilent) as described above. For each measurement, the activity was calculated after subtracting OAADPr autohydrolysis. The reactions were carried out in triplicate to calculate the kinetic parameters and their corresponding SDs were calculated. Commercial OAADPr (TRC Canada) was used as a standard.

### 4.4. De-mono-ADP-ribosylation assays

Human PARP1 E988Q mutant, an accepted model of mono(ADP-ribosyl)ated PARP1 substrate [31], was prepared using the hPARP1 kindly provided by J. M. Pascal (Thomas Jefferson University, PA, USA), as previously described [32]. For auto-ADP-ribosylation reactions, hPARP1 E988Q mutant (2.1 µM) was used in PARP reaction buffer (100 mM Tris–HCl pH 8.0, 10 mM MgCl_2_ and 1 mM DTT) in the presence of 200 µM β-NAD^+^ and 17 µg activated DNA during 1 h at 30°C. The reaction was then terminated with PARP1 inhibitor Rupacarib (5 µM), and 6 µM OiMacroD WT, N30A, G37V or D40A was added and incubated at 25°C for 30 min for de-mono-ADP-ribosylation. Reactions were terminated by placing them on ice. The released ADPr or ADPr standard (Sigma-Aldrich) was purified by ultrafiltration over Amicon Ultracel-3 columns, as previously described [10]. The experiments were carried out by triplicate.

Samples were analysed on an HPLC-MS equipment consisting of an Agilent 1100 Series HPLC coupled to an Agilent Ion Trap XCT Plus Mass Spectrometer using an electrospray (ESI) interface. Each sample (40 µl) was subjected to reversed-phase liquid chromatography on a Discovery C18 5 µm, 100 × 2.1 mm HPLC column (Supelco) and eluted at 0.2 ml min^−1^ with a mobile phase A (0.05% TFA in MilliQ water) and B (0.05% TFA in acetonitrile) 90 : 10 (v/v) for 10 min followed by a linear gradient from 10% to 100% solvent B in 20 min. The column was equilibrated with the starting composition of the mobile phase for 10 min before each analytical run. The mass spectrometer was operated in the positive mode with a capillary spray voltage of 3.5 kV. Data were obtained in the MS and MS/MS mode using Multiple Reaction Monitoring (MRM) and processed using the DataAnalysis program for LC/MSD Trap v. 3.2 provided by the manufacturer. ADPr was detected as the [M + H]^+^ ion at 560 *m/z* and confirmed with the transition 560 > 348 *m/z*.

### 4.5. Crystallization and data collection

The sparse matrix vapour diffusion sampling procedure [34] was used for screening crystallization conditions (4°C) in sitting drops of 0.5 µl reservoir fluid and 0.5 µl of 14 mg ml^−1^ OiMacroD in 20 mM HEPES pH 7.5. The best WT and G37V crystals were grown in 0.2 M ammonium sulphate, 0.1 M MES pH 6.5 and 30% (w/v) PEGMME 5 K. D40A and N30A mutant crystals were grown in 0.2 M magnesium formate with 20% (w/v) PEG3350 or 20% (w/v) PEG1500 with 20% glycerol, respectively. Complexes with ligands were obtained by soaking crystals in the same buffer supplemented with 20 mM ADP or ADPr, but removing MES when present. Crystals were flash-cooled in liquid nitrogen using the mother liquor supplemented with 15–30% glycerol. N30A mutant was directly frozen. Data were collected at XALOC beamline (ALBA Synchrotron, Barcelona) (*λ* = 0.979 Å) at 100 K (Oxford cryosystems) using a Pilatus 6M detector and processed using XDS, AIMLESS and TRUNCATE [35,36] (table 1). WT and D40A mutant crystals revealed a monoclinic (*P*2_1_) and orthorhombic (*P*2_1_2_1_2_1_) space group, respectively, with 2 subunits in the asymmetric unit (a.u.) (51–55% solvent). G37V and N30A mutants showed cubic (I*23*) and orthorhombic (*P*2_1_2_1_2_1_) space groups, respectively, with 1 subunit per a.u. (55% and 46% solvent, respectively) (table 1).

### 4.6. Model building and refinement

Phases were determined by molecular replacement with PHASER [35] using as search model the *E. coli* putative phosphatase (PDB 1SPV) devoid of ligands and water molecules. Rigid body refinement was performed stepwise with increased resolution, followed by automated refinement using REFMAC [35] or Phenix suite [37] and alternating with graphic manual model adjustment sessions with COOT [38]. ED from the resulting difference maps was interpreted, and a model was constructed for the complete polypeptide chain. In the case of N30A mutant, 11 well-ordered residues belonging to the His_6_-tag were included in the final model. B-factors and positional non-crystallographic symmetry restraints were used and gradually released as refinement progressed. All the diffraction data available were used for the refinement except in the calculation of *R*_free_, when 5% of the data were randomly selected. Crystal twinning was detected in all three WT OiMacroD crystals (twin fraction 0.35) and was refined by including the twinning correction option available in REFMAC5 (v. 5.6.0117). The final models of the WT enzyme with MES, ADPr and ADP at 1.9, 1.77 and 1.75 Å resolution, respectively, and those of the mutants at 1.35 (G37V), 1.75 (N30A) and 2.0 Å (D40A) exhibited excellent *R*_factor_/*R*_free_ values (table 1) and included one or two subunits (residues 1–185) per a.u. Structure analysis with RAMPAGE showed 96–97.3% of the residues in favoured regions of the Ramachandran plot for all the structures without outliers [39].

Structure superposition and r.m.s.d. calculation were performed with the SSM option of COOT [40] using default parameters. Figures were drawn using PyMOL (http://www.pymol.org/).

### 4.7. Deposition of coordinates and structure factors

The atomic coordinates and structure factors have been deposited in the Protein Data Bank with the following codes: OiMacroD-MES (5FUD), OiMacroD-ADPr (5L9K), OiMacroD-ADP (5L9Q), OiMacroD G37V (ADPr) (5LAU), OiMacroD D40A (5LCC) and OiMacroD N30A (5LBP).

### 4.8. Protein thermal shift assay

Protein melting curves to determine protein stability were obtained in the presence of the fluorescent dye SYPRO Orange (Molecular Probes). Thermal shift assays were carried out in triplicate in a medium containing 10× Sypro Orange (emission at 530 nm and excitation at 490 nm), 9 µg of purified OiMacroD or its mutants, and different ligands (AMP, ADP, ADPr or OAADPr) in 50 mM sodium phosphate pH 7.0, using a 7500 RT–PCR system (Applied Biosystems), as previously described [20].

### 4.9. *In silico* analysis

Phylogenetic analysis was carried out using Mega 7.0 with a ClustalW alignment and a neighbour-joining tree analysis [41]. Conserved blocks were determined using WebLogo [42] and the MacroD-like macrodomain sequences used in the phylogenetic tree, except for the unknown-function GDAP2 proteins.

## Supplementary Material

Supplementary Material
