# Supplementary material for: Structural and functional analysis of Oceanobacillus iheyensis macrodomain reveals a network of waters involved in substrate binding and catalysis
Source: Open Biol. 2017 Apr 26;7(4):160327. doi: 10.1098/rsob.160327 (PMC5413906; doi:10.1098/rsob.160327)
Supplement: Supplementary Material [file rsob160327supp1.pdf]

Supplementary Material

Structural and functional analysis of *Oceanobacillus iheyensis* macrodomain reveals a network of waters involved in substrate binding and catalysis

Authors:
Rubén Zapata-Pérez[†,⊥], Fernando Gil-Ortiz*[,‡,⊥], Ana Belén Martínez-Moñino[†], Antonio Ginés García-Saura, Jordi Juanhuix[‡], Álvaro Sánchez-Ferrer*[,†,§]

Affiliations:
†Department of Biochemistry and Molecular Biology-A, Faculty of Biology, Regional Campus of International Excellence "Campus Mare Nostrum", University of Murcia, Campus Espinardo, E-30100 Murcia, Spain

[‡]CELLS-ALBA Synchrotron Light Source, 08290 Barcelona, Spain

[§]Murcia Biomedical Research Institute (IMIB-Arrixaca), 30120 Murcia, Spain

[⊥]These authors contributed equally to this work.
*Correspondence and requests for materials should be addressed to. F.G.-O. (email: fgil@cells.es) or A.S-F (email: alvaro@um.es)

CONTENTS:

Table S1. B-factors of the protein and structural water molecules found in OiMacroD WT, mutants and related structures.
Table S2. Accession codes and organisms used in Figure S2.
Supplementary Figure S1. OiMacroD purification.
Supplementary Figure S2. Unrooted phylogenetic tree illustrating evolutionary relationships between OiMacroD and related macrodomains.
Supplementary Figure S3. Multiple sequence alignment between OiMacroD and related macrodomains
Supplementary Figure S4. Replacement of W5 in OiMacroD by residue Ser302 in the second binding macrodomain of hPARP15.
Supplementary Figure S5. F166 active and inactive conformations
Supplementary Figure S6. Stereo view of the dual conformer of the loop β6-α4.
Supplementary Figure S7. Dual ADP conformer found in the active centre of the OiMacroD-ADP crystal structure.
Supplementary Figure S8. Purity and identity of enzymatically synthesized OAADPr.

Table S1. B-factors of the protein and structural water molecules found in OiMacroD WT, its mutants and related structures.

B-factors	**Protein Overall B-factor**	W1	W2	W3	W4	W5	W6
MES	16.0	10.9	13.6	17.9	15.9	6.6	-
ADP-ribose	24.0	14.0	16.0	-[a]	17.4	16.1	16.8[b]
ADP	19.6	10.7	11.8	17.5	19.2	20.7	-
G37V	18.4	27.2	35.7	28.7	13.1	10.8	-
D40A	42.8	38.4	43.4	44.9	35.7	43.6	-
N30A	15.4	6.4	6.9	27.5	12.7	8.6	-
2X47	12.3	17.7	15.9	13.9	18.3	19.9	-
2BFQ	23.4	15.5	16.8	31.0	16.1	15.0	28.9
4IQY	20.8	13.1	17.0	16.9	10.6	11.0	13.6
3GPO	19.2	18.0	29.7	-[c]	16.7	15.8	

[a] W3 is absent in the OiMacroD-ADPr complex and is replaced by a glycerol molecule from the cryoprotectant, which carries out the same interactions.
[b] When an oligomer is present in the asymmetric unit, the data shown correspond to the average between all subunits
[c] W3 is absent in PDB 3GPO due to the presence of the side chain of residue W148, which hampers its binding. This residue shows low conservation and in other related structures is replaced by residues with shorter side chains that allow W3 binding. The O3´´-ribose atom in this structure interacts directly with residue T111.

Table S2. Accession codes and organisms used in Figure S2

Accesion code	Organism and protein designation
4GUA	Alphavirus P23 polyprotein
XP_003388308	*Amphimedon queenslandica* LOC100640713
XP_003386606	*Amphimedon queenslandica* OARD1
Q94JV1	*Arabidopsis thaliana* GDAP2
NP_030605	*Arabidopsis thaliana* macrodomain
NP_001077989	*Arabidopsis thaliana* PARG1
NP_850175	*Arabidopsis thaliana* PARG2
O28751	*Archaeoglobus fulgidus* 1521
Q3B7N1	*Bos taurus* ALC1
Q1LZ74	*Bos taurus* OARD1
F1ML90	*Bos taurus* PARP14
AHY61336	BtVs-BetaCoV/SC2013 ORF1ab polyprotein
Q59Z77	*Candida albicans* Ca019.2285
3GPO	Chikungunya virus (CHIKV) macrodomain
Q6PHJ5	*Danio rerio* MacroD2
Q1RLR9	*Danio rerio* Macro-H2A.1
NP_001020673	*Danio rerio* Macro-H2A.2
NP_001018591	*Danio rerio* OARD1
XP_687541	*Danio rerio* PARG
XP_001340167	*Danio rerio* PARP9b
1SPV	*Escherichia coli* YmdB
G3S362	*Gorilla gorilla* PARP14
Q86WJ1	Human ALC1
3EJG	Human coronavirus 229E macrodomain
2VRI	Human coronavirus NL63 NSP3 X-Domain
Q9NXN4	Human GDAP2
2X47	Human MacroD1
4IQY	Human MacroD2
O75367	Human Macro-H2A.1
Q9P0M6	Human Macro-H2A.2
Q9Y530	Human OARD1
NP_003622	Human PARG
Q460N5	Human PARP14b
Q460N3	Human PARP15
XP_005247877	Human PARP9b
D1YK16	*Lactobacillus gasseri* YmdB
YP_009047215	MERS-CoV NSP3 protein
Q9CXF7	*Mus musculus* ALC1
Q922B1	*Mus musculus* MacroD1
Q8R5F3	*Mus musculus* OARD1
V5IN46	*Neurospora crassa* macrodomain
Q8EP31	*Oceanobacillus iheyensis* macrodomain
YP_001949930	*Ralstonia* phage tail assembly-like protein
NP_013805	*Saccharomyces cerevisiae* S288C
AAS56031	*Saccharomyces cerevisiae* YBR022W
2FAV	SARS-CoV virus macrodomain
P67344	*Staphylococcus aureus* SAV0325 macrodomain
2XD6	*Thermus thermophilus* macrodomain
3GQO	Venezuelan Equine Encephalitis Virus (VEEV) Macrodomain

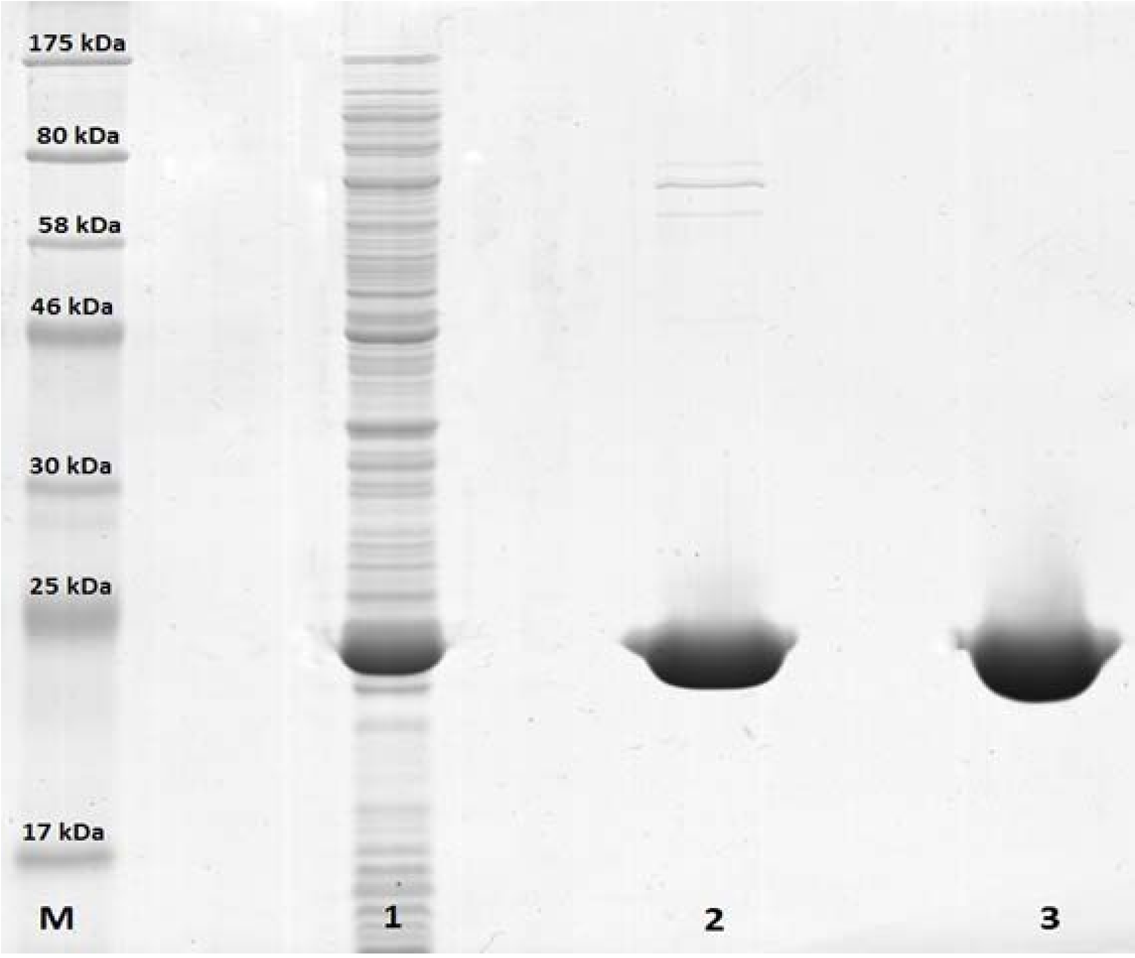

Supplementary Figure S1. OiMacroD purification. Each lane of the SDS-PAGE gel contains 10 µg protein. M: Molecular weight standards (NEB: P7708S). Lane 1: Cell extract after disruption and DNase treatment. Lane 2: Desalted HisTrap fraction. Lane 3: HiLoad Superdex fraction

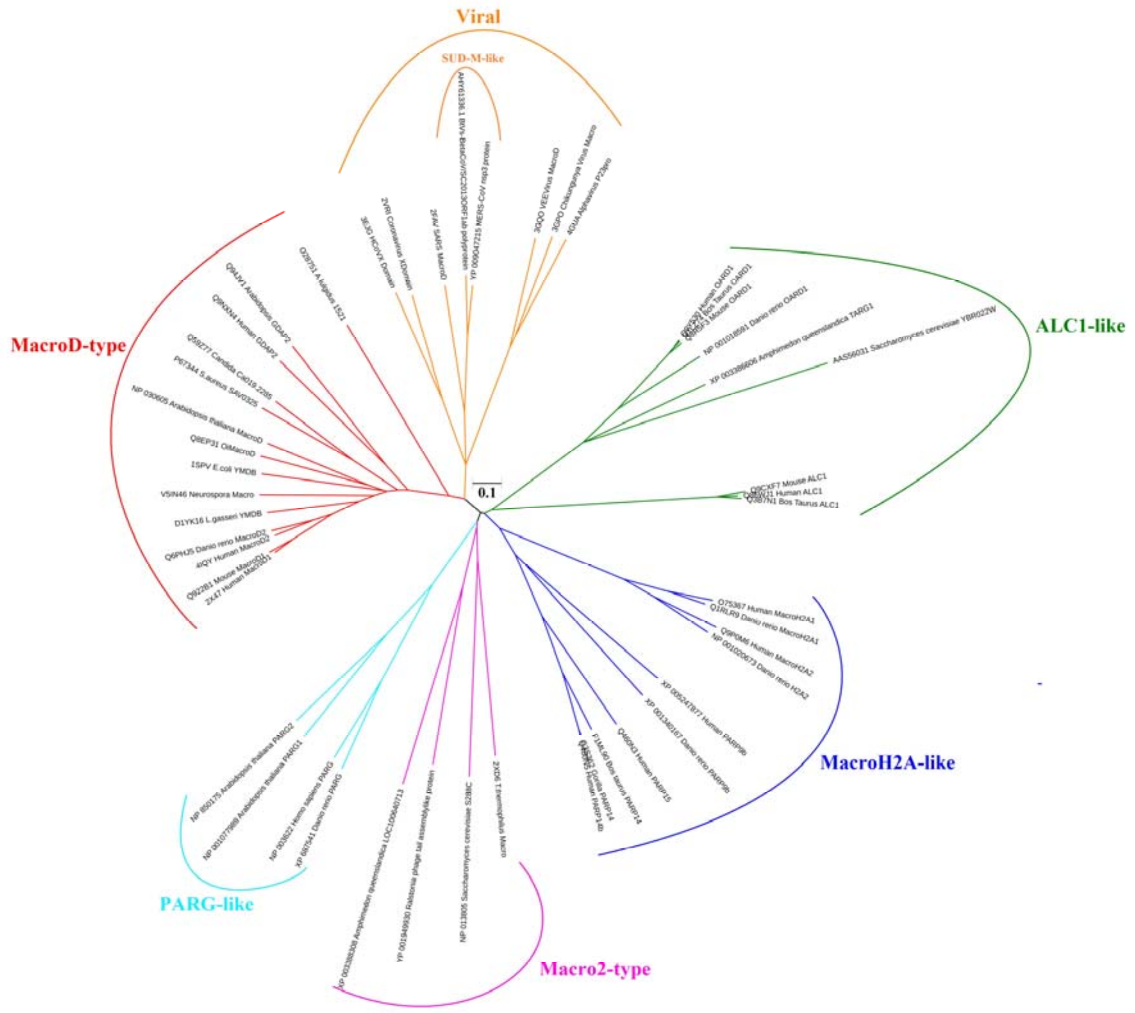

Supplementary Figure S2. Unrooted phylogenetic tree illustrating evolutionary relationships between OiMacroD and related macrodomains. Sequences selected for generating the tree are summarized in table S2.

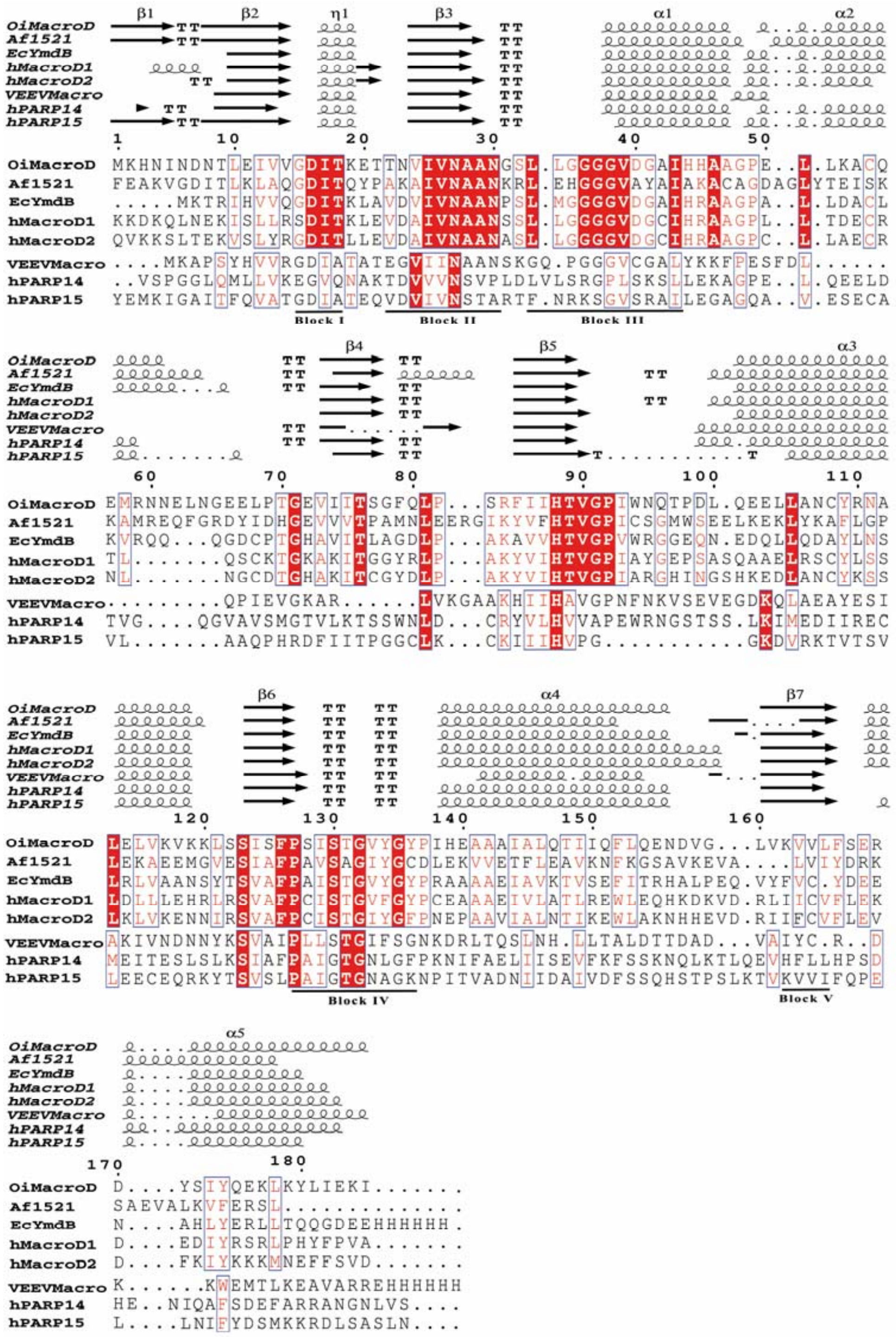

Supplementary Figure S3. Multiple sequence alignment between OiMacroD and related macrodomains. Strictly conserved residues have a red background and similar residues are marked with a blue square. Springs and arrows represent helices and strands of the secondary structure, respectively. Strict β-turns are represented as TT. Conserved blocks are underlined.

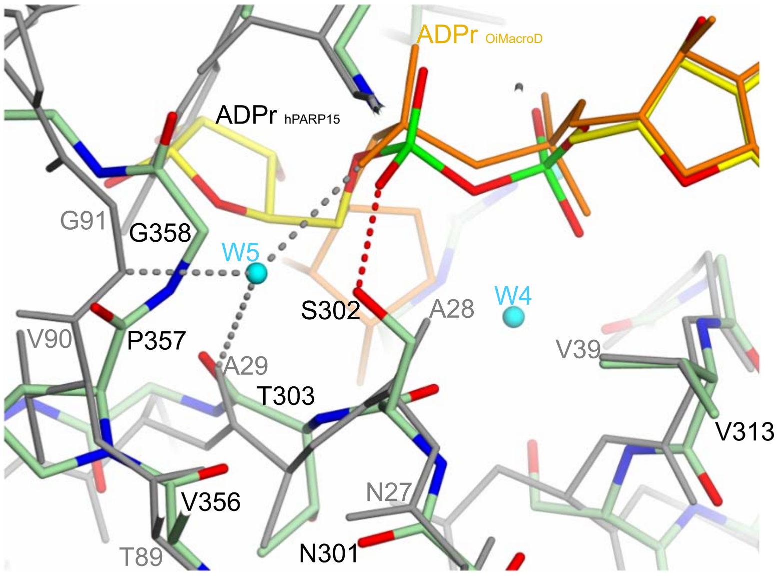

Supplementary Figure S4. Replacement of W5 in OiMacroD by residue Ser302 in the second binding macrodomain of hPARP15. Protein residues in hPARP15 and OiMacroD are coloured in green and grey, respectively. The ADPr molecule is in yellow (hPARP15) or orange (OiMacroD). Interactions are represented as dashed lines in red (hPARP15) or grey (OiMacroD). Water molecules are shown in cyan

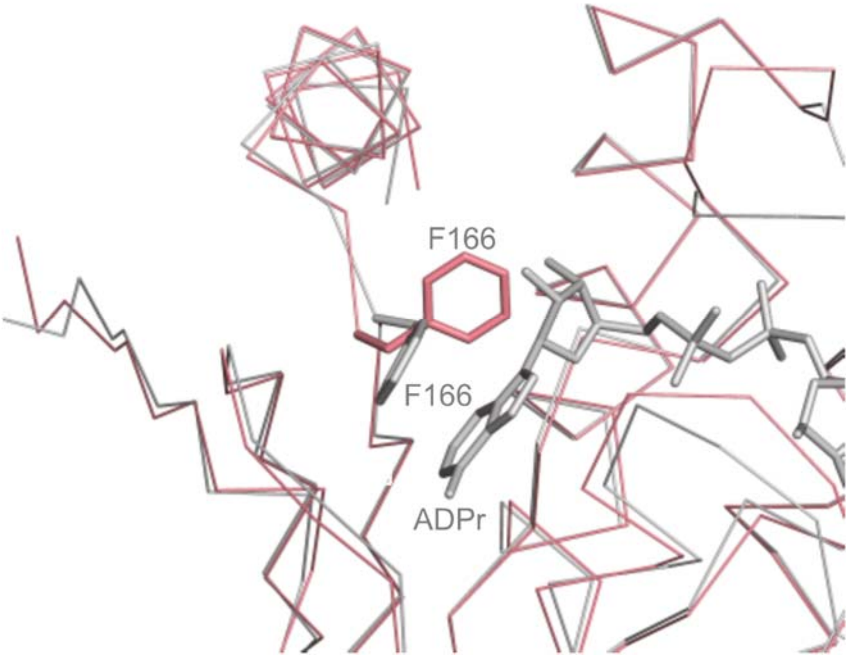

Supplementary Figure S5. F166 active and inactive conformations. Superimposition of mutant D40A (salmon) and ADPr complex (grey) crystal structures comparing the active (grey) and inactive (salmon) rotamer of residue F166. The ADPr molecule is shown in grey.

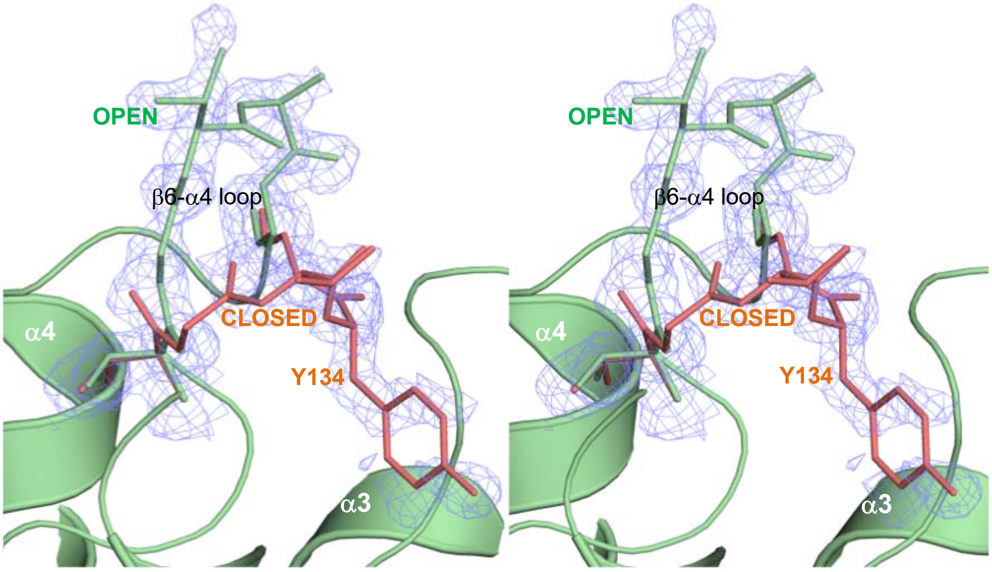

Supplementary Figure S6. Stereo view of the dual conformer of the loop β6-α4 as found in a subunit of the crystal structure of mutant N30A. The *2Fo−Fc* ED map (1.0 σ; blue) is shown for the open conformer (green; occupancy 0.55) and for the closed conformer (salmon; occupancy 0.45). Residue Y134 is only observed in the closed conformation (salmon).

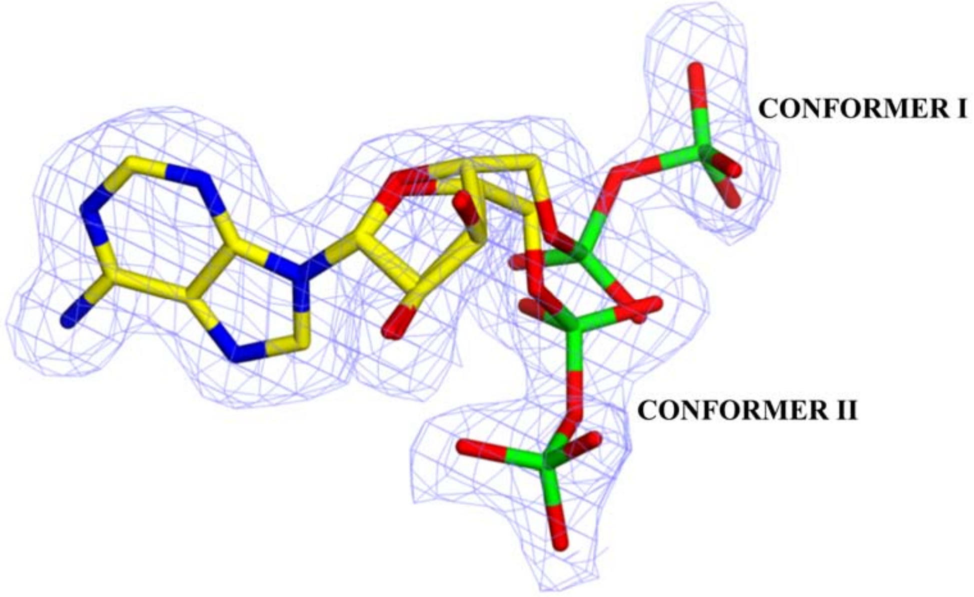

Supplementary Figure S7. Dual ADP conformer found in the active centre of the OiMacroD-ADP crystal structure (subunit A; 50% occupancy each). The *2Fo–Fc* ED map (1.0 σ; blue) is shown around the ADP molecule.

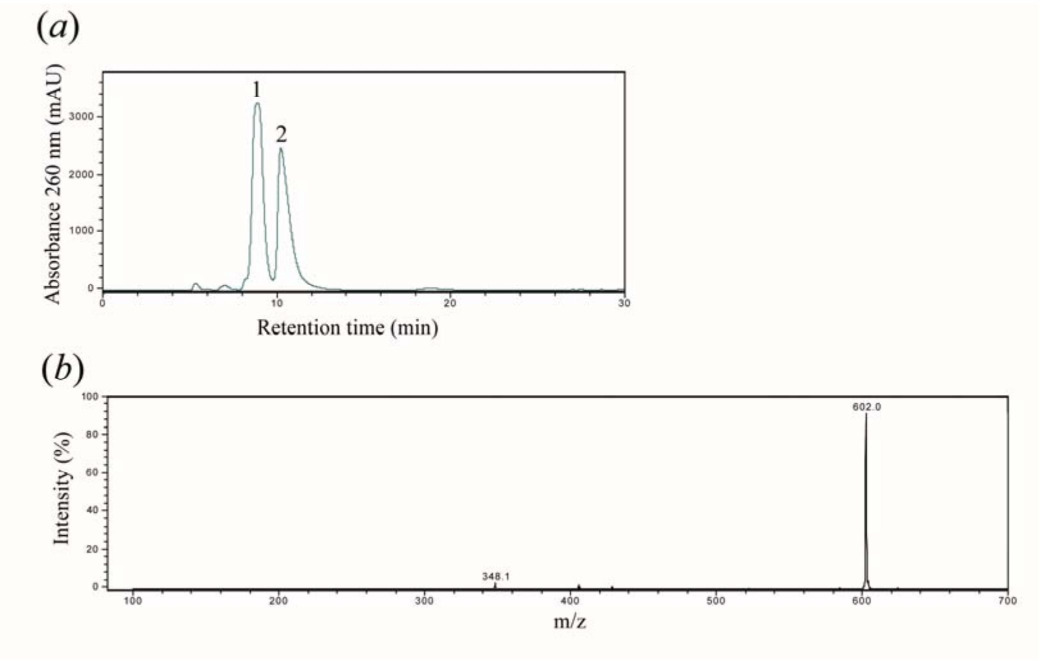

Supplementary Figure S8. Purity and identity of enzymatically synthesized OAADPr. (*a*) HPLC chromatogram at 260 nm showing the 3' (peak 1) and 2' (peak 2) isomers of OAADPr. (*b*) MS analysis of both OAADPr peaks. The 602.0 *m/z* peak corresponds to the expected mass for this compound.
